# Triglyceride-raising *APOA5* genetic variants are associated with obesity and non-HDL-C in Chinese children and adolescents

**DOI:** 10.1186/1476-511X-13-93

**Published:** 2014-06-05

**Authors:** Wei-fen Zhu, Chun-lin Wang, Li Liang, Zheng Shen, Jun-fen Fu, Pei-ning Liu, Lan-qiu Lv, Yi-min Zhu

**Affiliations:** 1 Department of Pediatrics, The First Affiliated Hospital, College of Medicine, Zhejiang University, 79 Qingchun Road, Hangzhou 310003, China; 2 Department of Central Laboratory, Children’s Hospital of Zhejiang University School of Medicine, Hangzhou, China; 3 Department of Endocrinology, Children’s Hospital of Zhejiang University School of Medicine, Hangzhou, China; 4 Department of Child Health Care, The Affiliated Yuying Children’s Hospital of Wenzhou Medical University, Wenzhou, China; 5 Department of Child Health Care, Ningbo Women & Children’s Hospital, Ningbo, China; 6 Department of Epidemiology & Biostatistics, Zhejiang University School of Public Health, Hangzhou, China

**Keywords:** Apolipoprotein A5, Obesity, Dyslipidemia, Children

## Abstract

**Background:**

Although the association between the apolipoprotein A5 (*APOA5*) genetic variants and hypertriglyceridemia has been extensively studied, there have been few studies, particularly in children and adolescents, on the association between *APOA5* genetic variants and obesity or non-high-density lipoprotein cholesterol (non-HDL-C) levels. The objective of this study was to examine whether *APOA5* gene polymorphisms affect body mass index (BMI) or plasma non-HDL-C levels in Chinese child population.

**Methods:**

This was a case–control study. Single nucleotide polymorphisms (SNPs) were genotyped using Matrix-Assisted Laser Desorption/Ionization Time of Flight Mass Spectrometry for an association study in 569 obese or overweight and 194 healthy Chinese children and adolescents.

**Results:**

Genotype distributions for all polymorphisms in both cohorts were in accordance with the Hardy-Weinberg distribution. The frequencies of the risk alleles in rs662799 and rs651821 SNPs in *APOA5* gene were all increased in obese or overweight patients compared to the controls. After adjusted for age and sex, C carriers in rs662799 had a 1.496-fold [95% confidence interval (CI): 1.074-2.084, *P* = 0.017] higher risk for developing obesity or overweight than subjects with TT genotype, while C carriers in rs651821 had a 1.515-fold higher risk than subjects with TT genotype (95% CI: 1.088-2.100, *P* = 0.014). Triglyceride (TG) and non-HDL-C concentrations were significantly different among rs662799 variants and both were higher in carriers of minor allele than in noncarriers for TG (1.64 ± 0.96 vs. 1.33 ± 0.67 mmol/L) (*P* < 0.001), and for non-HDL-C (3.23 ± 0.92 vs. 3.02 ± 0.80 mmol/L) (*P* = 0.005), respectively. There was also a trend towards increased TG and non-high-density lipoprotein cholesterol levels for rs651821 C carriers (*P* < 0.001 and *P* = 0.002, respectively). Furthermore, to confirm the independence of the associations between *APOA5* gene and TG or non-HDL-C levels, multiple linear regression analysis was performed and the relationships were not eliminated by adjustment for age, sex and BMI.

**Conclusions:**

These findings suggest the TG-raising genetic variants in the *APOA5* gene may influence the susceptibility of the individual to obesity, which may also contribute to an increased risk of high non-HDL-C levels in Chinese obese children and adolescents.

## Background

Obesity is one of the most complex clinical syndromes facing children, almost one-third of obese children remain obese in adulthood and that the risk of obesity as an adult was close to twice as high for obese children compared with those who had the normal weight during the growing period [1]. One of the most deleterious metabolic disorders of obesity is dyslipidemia, which is frequently occured and highly atherogenic. Recently, there is a growing consensus that non-high-density lipoprotein cholesterol (non-HDL-C), an indicator of dyslipidemia, appears to be more predictive of persistent dyslipidemia than total cholesterol (TC), low-density lipoprotein cholesterol (LDL-C) or high density lipoprotein cholesterol (HDL-C) for children [2]. In 2011, Expert Panel on Integrated Guidelines for Cardiovascular Health and Risk Reduction in Children and Adolescents released its summary report, which recommends non-HDL-C as a predictor of cardiovascular disease risk [3]. It appears that health professionals shall give more attention on non-HDL-C for a better care of children and adolescents, especially obese individuals.

In children and adolescents, the effects of environmental factors such as smoking, drinking and exercise are considerably weaker than in adults. According to the previous work the heritability of obesity in infancy is 60-80% and is reported to be 77% in preadolescents [4,5]. Therefore, it is crucial to investigate the genetic contribution to obesity and obesity-associated metabolic abnormalities, permitting screening and preventive treatment for obese children and adolescents. Many researchers have discovered multiple commonvariations that may give rise to genetic susceptibility of common childhood obesity, such as fat mass and obesity associated gene, melanocortin 4 receptor gene, glucosamine-6-phosphate deaminase 2 and so on [6-8]. Apolipoprotein A5 (*APOA5*) gene, located on chromosome 11 adjacent to *APOA1/APOC3/APOA4* gene cluster with known functions in the metabolism of plasma lipids [9], also modulates the risk of obesity in a number of studies [10-12]. However, in Chinese children and adolescents, limited data are available about the effect of *APOA5* variants on childhood obesity and obesity-associated dyslipidemia such as elevated non-HDL-C levels. To detect additional common variants associated with obesity and non-HDL-C in Chinese child population, we conducted this multicenter study.

## Results

The demographic variables and laboratory data of cases (569 obese or overweight children) and controls (194 healthy individuals) are presented in Table 1. As expected, there were no differences for the matching criteria of age and sex between the two cohorts. Compared with data of control group, obese or overweight group had significantly elevated body mass index (BMI) z-score, TC, triglyceride (TG), LDL-C, non-HDL-C levels and lower HDL-C levels (all *P* < 0.001).

**Table 1:** Demographic and metabolic characteristics of the study participants

	**Control group (n = 194)**	**Obese or over-weight group (n = 569)**	***P***
Sex (M/F)	149/45	417/152	0.252
Age (years)	10.94 ± 2.78	10.76 ± 2.02	0.410
BMI z-scores	(-0.27) ± 0.52	3.19 ± 1.38	<0.001
SBP (mmHg)	93.9 ± 11.27	112.45 ± 14.46	<0.001
DBP (mmHg)	66.35 ± 7.64	68.50 ± 9.09	0.001
TC (mmol/L)	3.80 ± 0.51	4.37 ± 0.92	<0.001
TG (mmol/L)	0.77 ± 0.32	1.49 ± 0.85	<0.001
HDL-C (mmol/L)	1.50 ± 0.31	1.25 ± 0.31	<0.001
LDL-C (mmol/L)	1.94 ± 0.41	2.56 ± 0.71	<0.001
non-HDL-C (mmol/L)	2.30 ± 0.43	3.14 ± 0.87	<0.001
FPG	4.84 ± 0.48	5.06 ± 0.65	<0.001

BMI, Body mass index; SBP, systolic blood pressure; DBP, diastolic blood pressure, TC, total cholesterol; TG, triglycerides; HDL-C, high-density lipoprotein cholesterol; LDL-C, low-density lipoprotein cholesterol; Non-HDL-C, non-high-density lipoprotein cholesterol; FPG, fasting plasma glucose.

Overall, rs662799 and rs651821 SNPs were selected and genotyped in the present study. Genotype distributions for all polymorphisms in both cohorts were in accordance with the Hardy-Weinberg distribution (data not shown). The prevalence of rs662799 C allelic variant in *APOA5* gene was significantly elevated in obese or overweight group compared with control group (30.3% vs 23.7%, *P* = 0.013). Meanwhile, a significant elevation was observed in frequency of risk C allele in rs651821 for obese or overweight group (30.4% versus 24.0% for controls, *P* = 0.016). Table 2 shows the genotype distribution of the two polymorphisms in case and control population. Frequencies of the TT, TC, and CC genotypes in rs662799 were 48.8%, 41.9% and 9.3% in obese or overweight group, and 58.8%, 35.0% and 6.2% in control group, respectively, which were statistically different between the two groups (*P* = 0.047). Additionally, Frequencies of the TT, CT, and CC genotypes in rs651821 were also significantly different between obese or overweight group and control group (*P* = 0.048).

**Table 2:** **Distribution of genotypes for *APOA5 *** **SNPs in cases and controls**

**Gene**	**SNP**	**Genotype**	**Control group**	**Obese or overweight group**	***P***
*APOA5*	rs662799	TT	114 (58.8%)	277 (48.8%)	0.047
		TC	68 (35.0%)	238 (41.9%)	
		CC	12 (6.2%)	53 (9.3%)	
*APOA5*	rs651821	TT	113 (58.2%)	274 (48.2%)	0.048
		CT	69 (35.6%)	243 (42.8%)	
		CC	12 (6.2%)	51 (9.0%)	

We then employed logistic regression to test the effect of the two *APOA5* SNPs on the risk of obesity, the results of which are presented in Table 3. Carriers of the C allele (CC + TC) in rs662799 had a 1.471-fold higher risk for developing obesity or overweight [odd ratio (OR) =1.471, 95% confidence interval (CI): 1.058-2.046, *P* = 0.022] than subjects with TT genotype, while carriers of the C allele (CC + TC) in rs651821 had a 1.486-fold higher risk than subjects with TT genotype (OR = 1.486, 95% CI: 1.069-2.066, *P* = 0.018). These associations remained significant after controlling for age and sex (rs662799: adjusted OR 1.496, 95% CI 1.074-2.084, *P* = 0.017; rs651821: adjusted OR 1.515, 95% CI 1.088-2.110, *P* = 0.014).

**Table 3:** **OR for obese or overweight associated with minor allele of each SNP at *APOA5 *** **gene**

**SNP**	**Genotype**	**OR (95% CI)**	***P *** **value**	**Adjusted OR**^**a **^**(95% CI)**	***P *** **value**
rs662799	TT	1		1	
	CC + TC	1.471 (1.058-2.046)	0.022	1.496 (1.074-2.084)	0.017
rs651821	TT	1		1	
	CC + TC	1.486 (1.069-2.066)	0.018	1.515 (1.088-2.110)	0.014

OR, odds ratio; CI, confidence intervals.

^a^Adjusted for sex and age.

**Figure 1:**
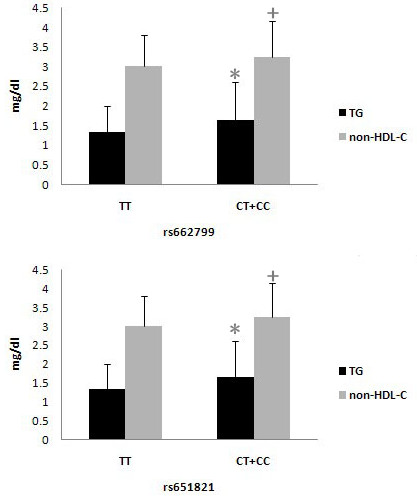
**Lipid profiles according to *APOA5 *rs662799 and rs651821 genotypes in obese or overweight children.** TG (black bar), non-HDL-C (gray bar). Results are expressed as mean ± SD and significant difference: *P < 0.05 non-risk genotypes vs risk genotypes (TG levels); ^+^*P <* 0.05 non-risk genotypes vs risk genotypes (non-HDL-C levels).

Next, the association between *APOA5* variants and obesity-related traits in obese or overweight children were examined. Figure 1 presents the changes in TG and non-HDL-C levels depending on the rs662799 and rs651821 polymorphism. Our results provided direct evidence for an association between the *APOA5* polymorphism and TG levels in Chinese children and adolescents. Also, non-HDL-C was influenced significantly by *APOA5* gene variants. We noted that the non-HDL-C in obese or overweight children with non-TT (CC + TC) genotype was 3.23 ± 0.92 mmol/L, which was elevated than that in obese children with TT genotype in rs662799 (3.02 ± 0.80 mmol/L) with a significant difference (*P* = 0.005). There was also a significant trend towards increased non-HDL-C levels for *APOA5* rs651821 C carriers (TT genotype: 3.01 ± 0.80 mmol/L; non-TT genotype: 3.24 ± 0.92 mmol/L; *P* = 0.002).

To confirm the independence of the associations between *APOA5* gene polymorphisms and TG or non-HDL-C levels, multiple linear regression models, using transformed log-TG as the dependent variable and including both *APOA5* rs662799 and rs651821 variants and other lipid influencing factors (age, sex, BMI) as the covariates, were performed in obese or overweight children. Carriership for *APOA5* rs662799 risk allele was identified as a significant and independent predictor of both TG (standardized β-coefficient = 0.184; *p* < 0.001) and non-HDL-C variabilities (standardized β-coefficient = 0.179; *p* < 0.001). Similarly, carriership for *APOA5* rs661821 risk allele was a significant and independent predictor for both TG (standardized β-coefficient = 0.120; *p* = 0.004) and non-HDL-C variabilities (standardized β-coefficient = 0.132; *p* = 0.002) (Table 4).

**Table 4:** ***APOA5 *** **variant carriership in multiple linear regressions with TG or non-HDL-C as the dependent variable in obese or overweight subjects**

	***B***	**95% CI for *B***	**Standardized β-coefficient**	***P***
TG				
Carrier of *APOA5* rs662799 C allele	0.275	0.154-0.397	0.184	<0.001
Carrier of *APOA5* rs651821 C allele	0.304	0.166-0.441	0.179	<0.001
Non-HDL-C				
Carrier of *APOA5* rs662799 C allele	0.208	0.065-0.350	0.120	0.004
Carrier of *APOA5* rs651821 C allele	0.231	0.088-0.374	0.132	0.002

TG, triglyceride; Non-HDL-C, non-high-density lipoprotein cholesterol; CI, confidence intervals.

Log-transformed TG was used as the dependent variable; covariates included *APOA5* polymorphisms, age, sex and BMI.

## Discussion

The rising healthcare costs of obesity and obesity-associated health problems are sufficient to justify prevention as daily work of the pediatric clinicians. By recognizing genetic variants that predispose to obesity, pediatricians could classify obese children into subgroups that might respond to specific diets or physical activity regimes, drugs or surgeries. In this study, we selected the most extensively studied SNP in *APOA5* gene, tag SNP rs662799 in the promoter region and another SNP rs651821, located 3 bp upstream from the predicted start codon of *APOA5*, which is in linkage disequilibrium with the much studied rs662799. The two tightly linked SNPs both show high minor allele frequencies in Chinese population, our results may be generalizable to other ethnic child population which have similar minor allele frequencies.

Our available data supported the hypothesis that the TG-raising genetic variants in the *APOA5* gene may also have high risks for obesity in Chinese children and adolescents. In consistent with our results, Horvatovich et al. revealed that APOA5*2 haplotype (containing the minor alleles of rs662799, rs2072560 and rs2266788) confers susceptibility to development of obesity in pediatric patients [13]. Additionally, in Aberle’s study, at baseline, TT homozygotes had a lower BMI compared to carriers of the C allele, although without a statistical significance [14]. Until now much is learned about the biologic function of *APOA5*, however, little is known about the associations between *APOA5* variants and body fat composition. Thus, mechanisms underlying our findings still remain hypothetic, requiring further work to prove. One speculated mechanism is that the effect of *APOA5* gene variants on the risk of obesity might be attributed to this locus being in interaction with other obesity-risk genes, such as *APOA1*, *APOC3* and *APOA4* genes [15,16]. Since the *APOA1/C3/A4/A5* cluster occupies a restricted chromosomal region, phenotypic effects that appear to result from mutations in one member of the cluster might actually derive from allelic associations with functional variants in another [17]. Other potential mechanisms could be linked to lipoprotein lipase (LPL) activation by the variable effects from multiple novel *APOA5* variants, resulting in varying degrees of impaired lipolysis [18]. As obesity is a complex, multifactorial condition for which heritability estimates vary widely. It might be the case in the association of obesity with *APOA5* gene variants, as a result, further studies are needed to clarify this issue.

Although the association between *APOA5* genetic variants and hypertriglyceridemia has been extensively studied [19-21] and also replicated in our study, there have been few studies, particularly in children and adolescents, on the association between *APOA5* genetic variants and non-HDL-C levels. Furthermore, the relationships for TG or non-HDL-C were not eliminated by adjustment for age, sex and BMI, suggesting that this genetic factors act independently of age, sex or BMI. In consistent with our results, Hubacek et al. also suggested that the rs662799 variant in the *APOA5* gene could be a significant genetic determinant for plasma non-HDL-C levels [22]. So far as we know, there are scarce studies to provide some mechanism by which *APOA5* gene variants could affect non-HDL-C levels. It is known that *APOA5* protein accelerates plasma hydrolysis of TG-rich lipoproteins by activation of plasma LPL [23]. We speculate that abnormalities in LPL activity could directly lead to delayed hydrolysis of TG-rich lipoproteins and elevated levels of intermediate metabolites in plasma, such as intermediate density lipoprotein (IDL), VLDL and chylomicrons, which resulted in an elevation of plasma non-HDL-C levels. Certainly, the detailed description of this hypothetic mechanismis needed for more intensive investigation.

The current study has several limitations that should be noted. First, the interactions between gene-gene, gene-environment and even different polymorphic loci of the same gene may influence the biological effects of the polymorphisms of the genes. Second, for all the polymorphisms, the number of the available studies in some subgroups, such as the control group was small. Third, due to the cross-sectional nature of the study design, the causal relationships between gene variants and clinical disorders can’t be drawn. Thus, we hope to perform full longitudinal association analyses in larger samples and more candidate genes in the future prospective studies. Furthermore, studies in the other populations will be useful to confirm our results.

## Conclusions

In conclusion, we herein demonstrated the TG-raising genetic variants in the *APOA5* gene may influence the susceptibility of the individual to obesity, which may also contribute to an increased risk of high non-HDL-C levels in Chinese obese children and adolescents. We believe that our findings will improve the understanding of biological functions of *APOA5* gene variants.

## Methods and procedures

### Study population

This case–control study was conducted from 2011to 2012. We totally recruited 569 obese or overweight patients (“case”) at the age of 7–16 from outpatients who visited one of the following three medical centers for their weight problems: (1) Department of Endocrinology, Children’s Hospital of Zhejiang University School of Medicine, Hangzhou, Zhejiang; (2) Department of Child Health Care, the Affiliated Yuying Children’s Hospital of Wenzhou Medical University, Wenzhou, Zhejiang; (3) Department of Child Health Care, Ningbo Women & Children’s Hospital, Ningbo, Zhejiang. According to the diagnostic criteria developed by the Working Group for Obesity Task Force in China [24], the 85th and the 95th percentiles of age- and sex-specific BMI were used as cut-off points for defining overweight and obesity, respectively. In addition, we recruited 194 healthy children (“control”) who were ascertained at the Department of Child Health Care, Children Hospital of Zhejiang University School of Medicine. Exclusion criteria for the controls consisted of the known presence of diabetes, hypertension, dyslipidaemia, other endocrine metabolic or kidney diseases and the use of medications that altered blood pressure, glucose, or lipid metabolism.

Informed consent was obtained from the parents, and approved by the ethics committee of all the aforementioned three medical centers: the Children Hospital of Zhejiang University School of Medicine, the Affiliated Yuying Children’s Hospital of Wenzhou Medical University and Ningbo Women & Children’s Hospital.

### Physical parameters

Body height was measured to the nearest 0.1 cm, while weight was measured to the nearest 0.1 kg. At the time the measurements were made, the participants were only their underwear. BMI was calculated as body weight (kg) divided by the square of body height (m^2^). Age- and sex-specific BMI z-scores were used as continuous dependant variables for each model [25].

### Biochemical parameters

Baseline blood samples were obtained early in the morining after an overnight fast and immediately measured. Serum TC and TG were determined with an enzymatic colorimetric. Serum HDL-C and LDL-C were measured through a direct assay. All biochemical parameters were measured in a conventional automated analyzer (BECKMAN Synchron Clinical System CX4, American). Non-HDL-C was calculated as TC level minus HDL-C. The remaining blood samples were stored at -80 degrees Celsius for SNP genotyping.

### SNP genotyping

DNA was isolated from blood samples using Blood Genomic DNA Miniprep Kit (AxyPrep) according to the protocol of the manufacturer. All samples were originally genotyped using an automated platform MassARRAY (Sequenom, San Diego, CA).

DNA sequence containing the target SNP site was first amplified by polymerase chain reaction. By using the SNP specific primers, the products were extended one base in SNP sites. After applied into the MassARRAY SpectroCHIP array, the products were crystallized with matrix in the chip. And then the crystal containing chip was directed into the mass spectrometer vacuum tube and excited using an instantaneous nanosecond (10–9 s) laser. The molecular of matrix absorb the laser radiation, which resulted in energy accumulation causing crystal matrix sublimation, DNA molecule desorption and transformation to metastable ions [26].

### Statistical analysis

Quantitative variables were expressed as mean ± standard deviation (SD) and differences between groups were calculated by Student’s *t*-test. The chi-square test was employed to test for deviations of the genotypes from the Hardy-Weinberg equilibrium and to evaluate categorical variables. To determine whether SNP polymorphisms were independent modulators on the development of obesity, logistic regression analysis was performed adjusted for age and sex. We applied ANOVA and the Studens’s *t* test to compare crude means across genotype groups. A multiple linear regression models procedure was performed by using transformed log-TG as the dependent variable, and including carrierships of *APOA5* variants and other influencing factors (age, sex, BMI) as the covariates. The power calculation was performed using Quanto software (http://hydra.usc.edu/gxe/). Our study had ≥68.1% to ≥98.5% power (enrolment of 569 cases and 194 controls) to detect Ors of 1.50 to 2.00 for obesity under an additive model, assuming a significance of 0.05, an allele frequency of 0.267, and overweight or obesity prevalence of 19.9% in 7- to 16- year old Chinese Children [27]. Statistical analysis was performed using SPSS 17.0 software, and two tailed *P*-value <0.05 was considered statistically significant.

## Abbreviations

non-HDL-C: Non-high-density lipoprotein cholesterol; TC: Total cholesterol; LDL-C: Low-density lipoprotein cholesterol; HDL-C: High density lipoprotein cholesterol; APOA5: Apolipoprotein A5; BMI: Body mass index; TG: Triglyceride; OR: Odd ratio; CI: Confidence interval; LPL: Lipoprotein lipase.

## Competing interests

The authors have no competing interests.

## Authors’ contributions

Dr. WZ did the experiments and data analysis, and drafted the initial manuscript. Prof. LL designed thestudy, and critically reviewed and revised the manuscript. Dr. CW and Prof. JF also designed the study and participated in critical discussion. Dr. ZS mainly collected the data and participated in doing experiments. Dr. PL and Dr. LL both all contributed to the collection of data. Prof. YZ provided help for the data analysis and interpretation. All authors read and approved the final manuscript.

## Authors’ information

We all agree that every author is the co-first author of the paper.
